# Benchmarking the Performance of Mobile Laser Scanning Systems Using a Permanent Test Field

**DOI:** 10.3390/s120912814

**Published:** 2012-09-19

**Authors:** Harri Kaartinen, Juha Hyyppä, Antero Kukko, Anttoni Jaakkola, Hannu Hyyppä

**Affiliations:** 1 Department of Remote Sensing and Photogrammetry, Finnish Geodetic Institute, P.O. Box 15, FI-02431 Masala, Finland; E-Mails: juha.hyyppa@fgi.fi (J.H.); antero.kukko@fgi.fi (A.K.); anttoni.jaakkola@fgi.fi (A.J.); 2 Helsinki Metropolia University of Applied Sciences, FI-00079 Metropolia, Finland; E-Mail: hannu.hyyppa@gmail.com; 3 School of Science and Technology, Aalto University, FI-00076 Aalto, Finland

**Keywords:** GNSS/INS, reference data, systems, laser scanning, point cloud, accuracy, mobile, geometric

## Abstract

The performance of various mobile laser scanning systems was tested on an established urban test field. The test was connected to the European Spatial Data Research (EuroSDR) project “Mobile Mapping—Road Environment Mapping Using Mobile Laser Scanning”. Several commercial and research systems collected laser point cloud data on the same test field. The system comparisons focused on planimetric and elevation errors using a filtered digital elevation model, poles, and building corners as the reference objects. The results revealed the high quality of the point clouds generated by all of the tested systems under good GNSS conditions. With all professional systems properly calibrated, the elevation accuracy was better than 3.5 cm up to a range of 35 m. The best system achieved a planimetric accuracy of 2.5 cm over a range of 45 m. The planimetric errors increased as a function of range, but moderately so if the system was properly calibrated. The main focus on mobile laser scanning development in the near future should be on the improvement of the trajectory solution, especially under non-ideal conditions, using both improvements in hardware and software. Test fields are relatively easy to implement in built environments and they are feasible for verifying and comparing the performance of different systems and also for improving system calibration to achieve optimum quality.

## 1. Introduction

Mobile mapping is currently an emerging technology, which is used to enhance the attractiveness of mobile phone ecosystems by collecting large data sets covering the biggest cities. Automatic techniques to process the data into 3D models is a topic of increasing importance since accurate and intelligent up-to-date 3D roadside information will be needed in the future, especially for vehicle and pedestrian navigation and location-based services. Most mobile mapping systems are based on images taken from mobile systems, but the use of mobile laser scanning (MLS) together with images is also increasing. The advantage of laser scanning is in its high level of automation in creating the geometry for virtual models using point cloud data.

MLS is a multi-sensor system that integrates various navigation, laser scanning, and other data acquisition sensors on a rigid, moving platform (typically a van or a car) for acquiring road-side data. The navigation sensors typically include Global Navigation Satellite System (GNSS) receivers and an Inertial Measurement Unit (IMU), while the data acquisition sensors typically include terrestrial laser scanners and imaging systems.

Recent studies on MLS systems and their accuracy can be found in Barber *et al.* [1], Brenner [2], Clarke [3], El-Sheimy [4], Früh and Zakhor [5], Graham [6], Haala *et al.* [7], Hassan and El-Sheimy [8], Jaakkola *et al.* [9], Kukko [10], Kukko *et al.* [11–13], Kukko and Hyyppä [14], Lehtomäki *et al.* [15], Manandhar and Shibasaki [16], Petrie [17], Shen *et al.* [18], Steinhauser *et al.* [19], Tao and Li [20], Weiss and Dietmayer [21], Yu *et al.* [22] and Zhao and Shibasaki [23–25]. Mobile laser scanning systems are being developed both in the field of robotics and surveying. Relatively complete lists of MLS systems have been presented by Petrie [17] and Narayana [26].

The experiences gained in earlier remote sensing research have shown that permanent test fields with accurate ground truth are valuable tools for analyzing the performance of remote sensing systems and methods. To be able to compare various systems or methods, the test data should be from a common test field; this has also been demonstrated in an earlier EuroSDR project “Building Extraction” [27] and in the EuroSDR/ISPRS project “Tree Extraction” [28]. The task of comparing mobile laser scanning systems is challenging as the accuracy of the georeferenced point cloud is highly dependent on the GNSS visibility during data acquisition and the satellite geometry is constantly changing. Also, the seasons of the year can affect satellite visibility if there are tall deciduous trees close to the trajectory.

Prior to the present study, there have been only a few studies that have focused on MLS comprehensively in combination with test fields. System manufacturers have carried out and published their own tests, but only a few publications exist where system performance has been examined using an established test field and where the results have been analyzed by an independent actor.

Barber *et al.* [1] used RTK-GPS measurements to collect reference data on two test sites to validate the geometric accuracy of the Streetmapper MLS system. The main focus then was on elevation accuracy, while only a few control points, measured on white line markings on the road, were used for the analysis of planimetric accuracy.

Researchers of the University of California at Davis, United States, used total station and static TLS data to analyze the accuracy of MLS systems (Streetmapper 360, Optech Lynx and Ambercore Titan) when producing digital terrain models of pavement surfaces [29]. Then only elevation accuracy was the subject of concern.

Haala *et al.* [7] demonstrated that the StreetMapper system could produce dense 3D measurements with an accuracy level of 30 mm in good GNSS conditions. Furthermore, the remaining differences between the point clouds from different scanners, due to the imperfect boresight calibration of the upward looking scanner, could be corrected during post processing. Under degraded GNSS conditions, they reported a georeferencing error of up to 1 m for the horizontal position. They also reported that despite the limited absolute accuracy, 3D point measurements during bad GNSS conditions are still useful, especially if the purpose is mainly to exploit their relative positions. As an example, they presented that the standard deviation of such data is only 5 cm if the points from two scanners are combined and 2.6 cm if the points are separated for each scanner. Thus, such data are feasible for the extraction of the features of windows or passages if a certain error as to their absolute position is acceptable. Since the best laser systems in MLS are capable of estimating the range with an accuracy of 2 mm, and as direct georeferencing dominates in error propagation, an improvement is needed in the of georeferencing solution. The options in improving the georeferencing solution include more accurate calibration of the relative orientation of the MLS system components, automatic/manual detection of those objects (the position of which is known) from the road sides that may be used to improve georeferencing, and development of new data fusion approaches for MLS. The most in-depth analysis of MLS quality thus far is that presented by Haala *et al.* [7].

This paper concentrates on evaluating the geometrical properties of laser point clouds collected by various commercial and research-based MLS systems in good GNSS conditions on an established urban test field.

## 2. Benchmarking Mobile Laser Scanning Systems

### 2.1. Materials

#### 2.1.1. Test Field

**Figure 1.**
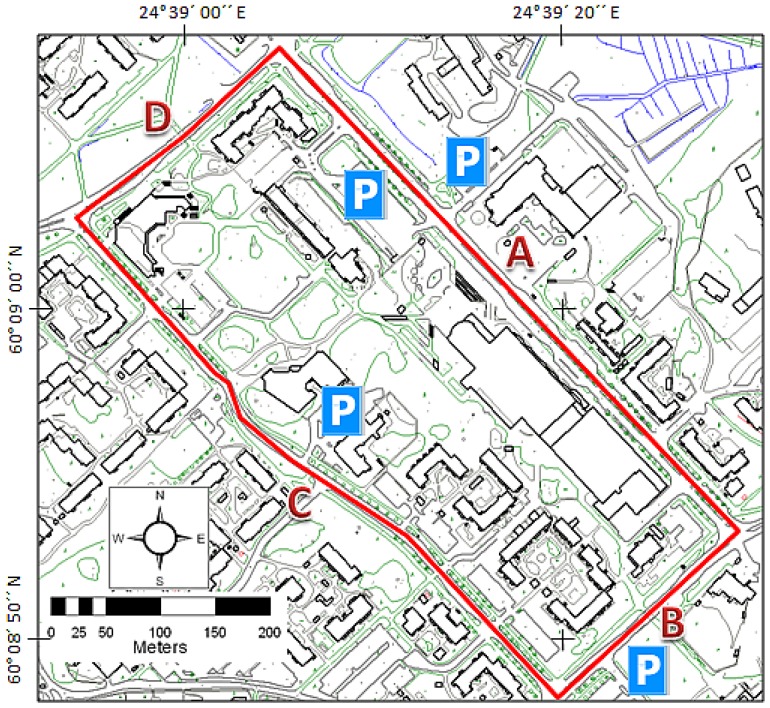
The Espoonlahti test field for mobile laser scanning covers 1,700 m of road environment. The driving route is marked by the red line, and the various sections are marked by the red letters A–D. The parking spaces are marked by the letter P. The map data were provided by the courtesy of the City of Espoo.

**Figure 2.**
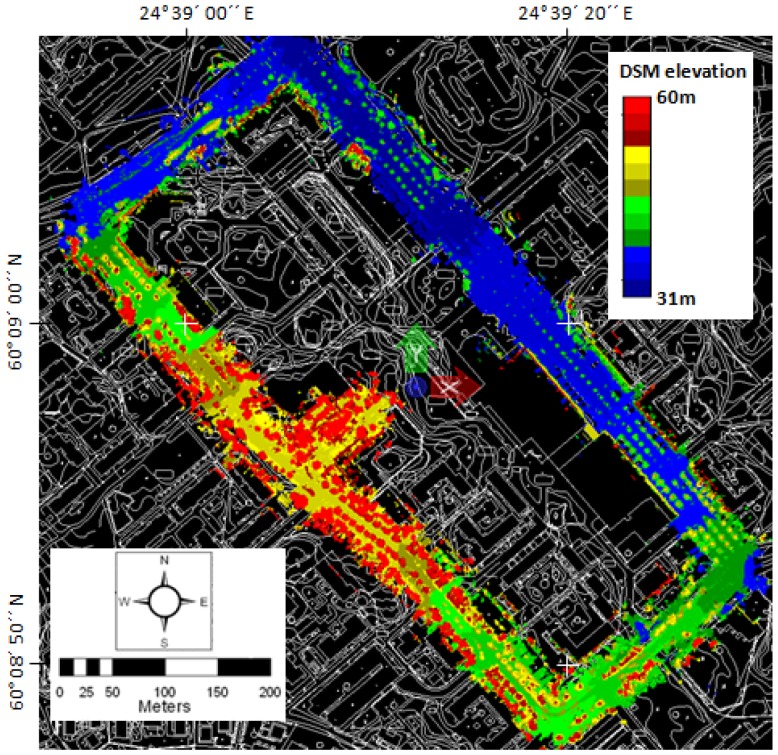
A digital surface model of the Espoonlahti test field (based on MLS data). The map data were provided by the courtesy of the City of Espoo.

The test field was implemented in Espoonlahti, about 16 km west of Helsinki. The test field covers one block around the Lippulaiva shopping mall covering 1,700 m of road environment (Figure 1). The test field was divided into four sections separated by intersections as shown in Figure 1. GNSS visibility from Sections A, B and D is good, although some trees and higher buildings may restrict the visibility of lower satellites. As can be seen from the digital surface model in Figure 2, Section C has large trees standing close to the road, thus making the GNSS conditions far more challenging. There are many types of buildings and other constructions, such as stairs and walls, in the area, as well as hundreds of pole type objects, such as lamp posts, traffic signs and trees. The road area, as well as most of the terrain close to the road, is very flat in Section A. The other sections are more variable in regard to terrain elevation, both on the road area and in the surroundings. The height difference between the lowest and highest points along the road is 12 m (Figure 2).

#### 2.1.2. Reference Data for Accuracy Evaluation

**Figure 3.**
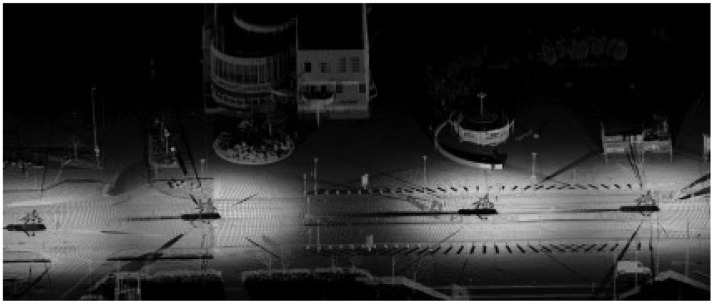
An example of reference point clouds. The scanning locations can be seen as shadows on the road that by the car was driven along.

Dense terrestrial laser scanner point clouds were used to obtain the reference targets for the analysis of geometric accuracy. The reference point clouds were collected on 7 May 2009 using FGI's mobile mapping system called the ROAMER [11], Road Environment Mapper in static mode. The ROAMER was installed on the roof of a car, and the car was kept standing static on the road during each 360° scanning performed using of the FARO Photon 80 terrestrial scanner (Figure 3). The scan resolution was set to 0.0013 rad point separation. The georeferencing of individual scannings was computed during post-processing: the scanner position and heading were obtained from the ROAMER's SPAN navigation system and the scannings were leveled using the scanner's built-in inclinometer. The virtual GPS reference station data used in GPS post-processing were downloaded from the GPSNet.fi service. The SPAN data were processed using the Waypoint Inertial Explorer software, which gave the estimated accuracies of 11 mm in 3D-position and 0.027° in heading (RMS) for the ROAMER's inertial measurement unit (IMU) during the measurements. The offset between the IMU and the scanner origin, as well as the offset between the SPAN and the scanner heading, were determined during system calibration.

The ROAMER's TLS/static data were validated against 150 check points measured using a total station (Trimble 5602S DR200+). A total of nine ground control points (GCPs) were measured for the total station setup around the Espoonlahti test field using repeated real-time GPS measurements (Leica SR530). Eight individual measurements were taken at each point using different reference data sources (RTK-GPS using its own reference station and VRS-GPS using a virtual reference station) and different satellite constellations (a few hours passing between the measurement sessions). For each GCP, the first two sessions were measured using RTK-GPS (with an expected accuracy of 1 cm + 1–2 ppm in horizontal plane and 1.5–2 cm + 2 ppm in height [30]), and then two sessions using VRS-GPS (with an expected accuracy of 2 cm in horizontal plane and 4 cm in height [31]). A new GPS initialization was acquired between each session. This procedure was carried out for a second time after a few hours. The GCP coordinates were then computed as a mean of the obtained eight coordinates. The maximum standard deviation of the eight “original” coordinates was 23 mm in horizontal plane and 30 mm in elevation (the averages were 13 mm and 20 mm, respectively).

With most of the ROAMER scannings, the comparisons showed that the check points and point clouds matched one another within the standard deviation of the GCPs, *i.e.*, a couple of centimeters, but with a few scans there was error in the leveling of the point cloud. In these cases there had been a passing bus visible in the scanning data, and so it is obvious that a large vehicle had caused a disturbance in the functioning of the scanner's inclinometer. These point clouds were then re-leveled by using neighboring point clouds and check points.

Following the point cloud validation, the targets for accuracy analysis were measured along a 350 m length of the test field's Section A (Figure 1) with the best GNSS visibility. The TerraScan-software by TerraSolid Ltd. was used for all point cloud operations. Firstly, the ground points were classified and a regular grid with a point spacing of 5 cm was computed to achieve an even distribution of the ground points. This grid was then thinned by selecting every 1,000th point, and these thinned points were compared to the original ground points. Every thinned point deviating more than 5 mm from the original data was deleted, and the remaining points were selected as the reference points for analysis of the elevation accuracy. The complete ground reference data for the elevation consisted of 3,283 points, and also the distance and direction to all possible driving trajectories were determined for these points.

**Figure 4.**
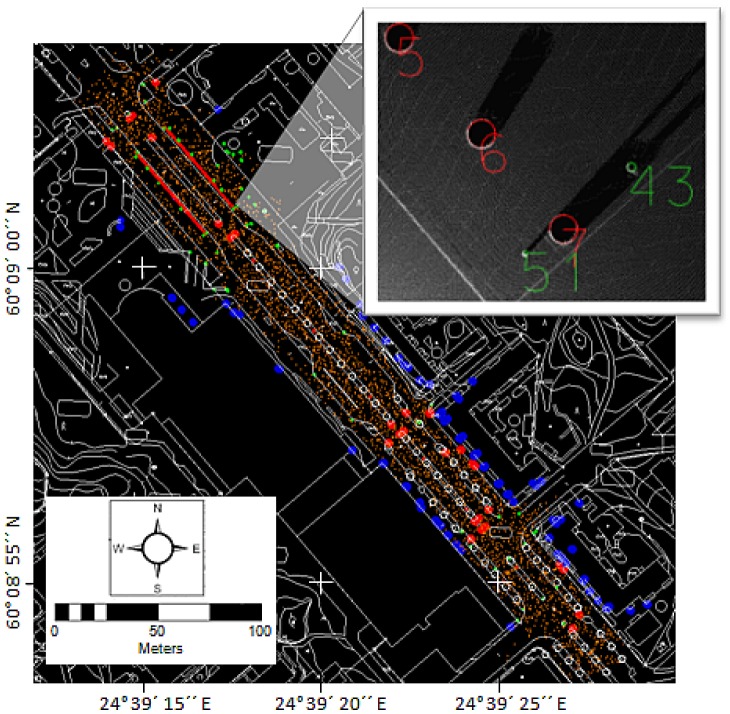
The reference targets: orange points are elevation reference points and blue (targets 5 m above ground), red (large poles) and green (small poles) points are planimetric reference points (the map data provided by the courtesy of the City of Espoo). The small figure in the top right corner shows a detail of the measured poles from close-to-ground laser points.

The ground reference data were used to separate all laser points within 10 cm below and 50 cm above the ground, and these close-to-ground points were then used to measure the reference targets for the evaluation of planimetric accuracy (Figure 4). The targets included centers of poles, building corners and curb corners. Another slice of laser points, 1 m thick, was taken at approximately 5 m above the ground, and these laser points were used to measure more building corners and centers of poles. Altogether 273 planimetric reference targets were measured. The pole coordinates were measured by visually fitting a circle to the point cloud in the top view, and the centre of the circle was used as the reference coordinate.

#### 2.1.3. Benchmarked Mobile Laser Scanning Data

Mobile laser scanning data were collected from the test field using five different systems (Tables 1 and 2). The test field was driven in both clockwise (CW) and counter-clockwise (CCW) direction at a speed of about 30–40 km/h with all of the systems.

**Figure 5.**
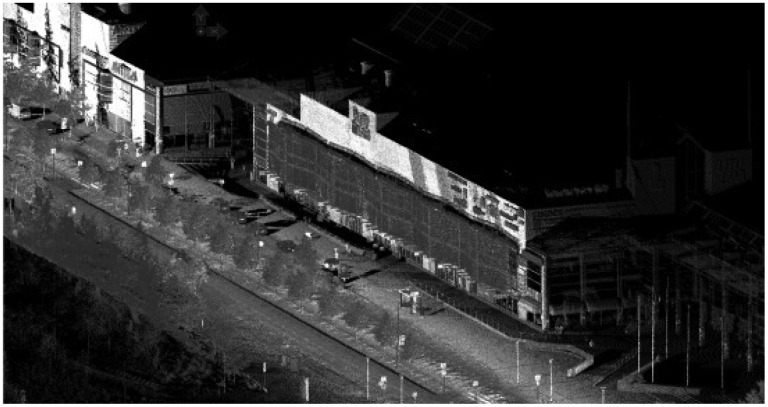
Optech Lynx data.

**Figure 6.**
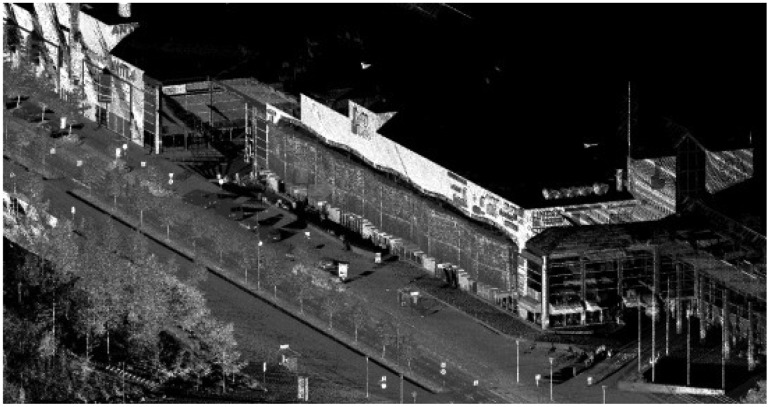
Streetmapper data.

**Figure 7.**
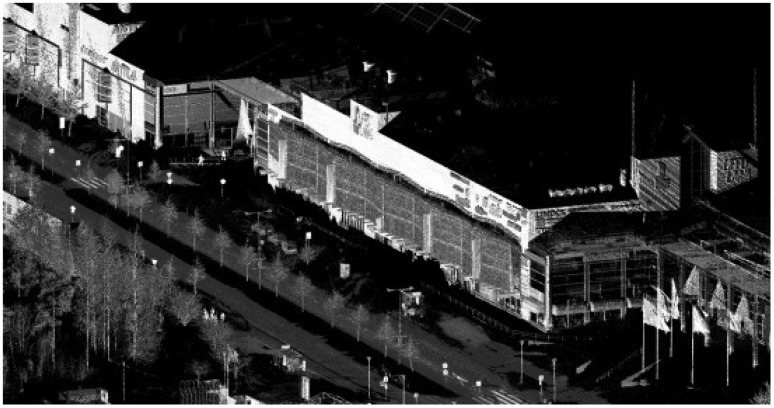
RIEGL VMX-250 data. There were still patches of snow on the ground during the data acquisition, and these resulted in voids in the data.

**Figure 8.**
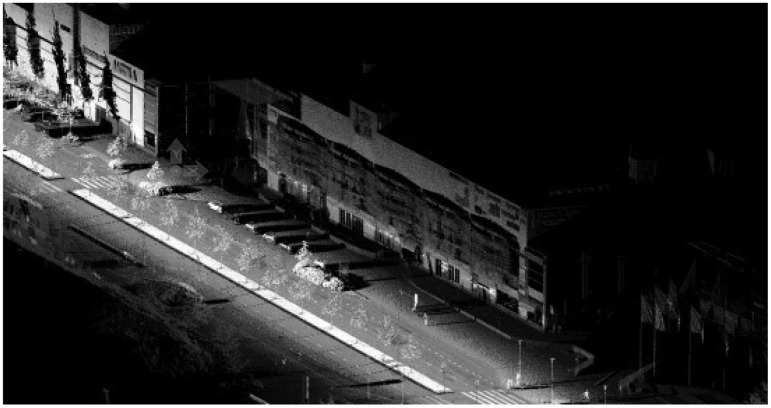
ROAMER data.

**Figure 9.**
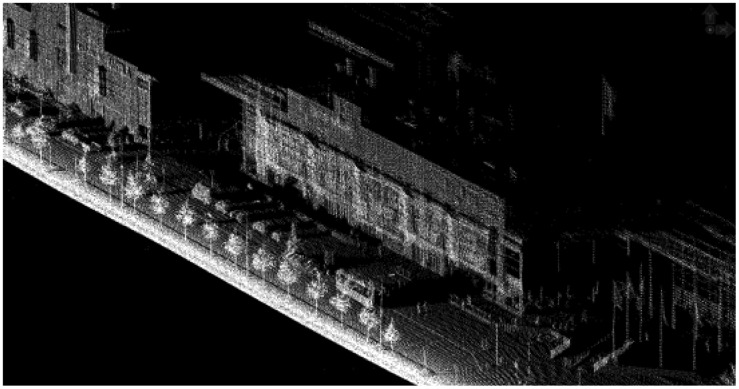
Sensei data. Due to the scanning angle, the Sensei covered only one side of the trajectory at a time.

Examples of the acquired point clouds are shown in Figures 5–9. The laser points are visualized by their intensity value, but with the exception that Sensei does not record intensity.

**Figure 10.**
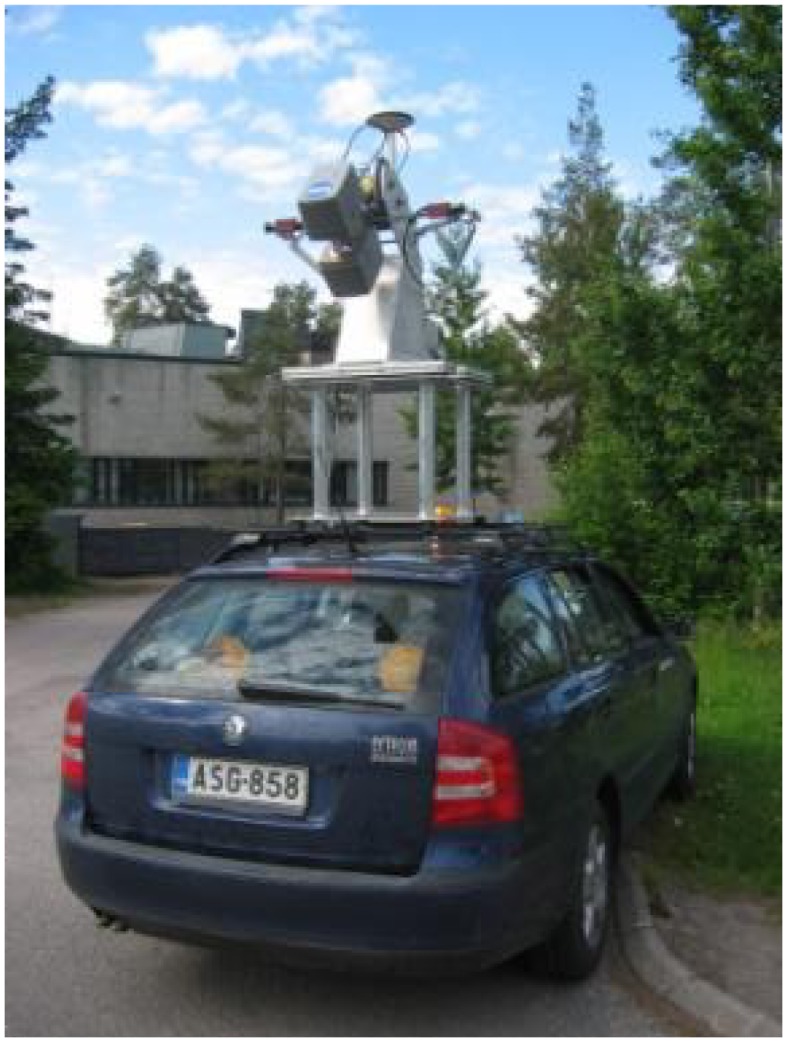
The ROAMER.

FGI's **ROAMER** system, developed in-house [11], has been operational since the summer of 2007. Mobile mapping data from test field using this system were acquired in June 2009. At that time, the ROAMER consisted of a FARO Photon 80 terrestrial laser scanner and a NovAtel SPAN positioning system (NovAtel DL-4 plus GPS-receiver, a NovAtel GPS-702-GG antenna and a Honeywell HG1700 AG58 inertial measurement unit (IMU) with ring laser gyros). Later on, the laser scanner was updated, and currently a FARO Photon 120 terrestrial laser scanner is used. The maximum point measurement rate of the Photon 80 scanner was 120 kHz and its range was 76 m (Photon 120: 976 kHz and 153 m, respectively). The laser profiling was carried out using a scanning frequency of 48 Hz. The ROAMER has an adjustable scanning angle, and in the Espoonlahti exercise the scanner was operated for measuring profiles by having it tilted 45° below the horizontal; see Figure 10. The ROAMER is the only system in this comparison, which utilized a laser scanner with continuous wave laser and phase-shift-based distance measurement. The beam size of the scanner was also the smallest in the test.

The direct georeferencing of the ROAMER point clouds was computed using the Waypoint Inertial Explorer™ GPS-IMU post-processing software. The GPS reference station data were acquired from the Finnish virtual reference station (VRS) network GPSNet.fi. After georeferencing, the dark points were deleted by filtering out points with intensity value of less than 8,000 (range 0–20,470), and isolated points were deleted by filtering out points that had less than 50 points within a 2 m radius around them. TerraScan by Terrasolid Ltd was used for the filtering.

Two point clouds were analyzed for the ROAMER. In the first one, the georeferencing of the point cloud was computed using the calibration values between the instruments determined only in laboratory calibration. The laboratory calibration was based on measuring the physical offsets and rotations between the scanner, IMU and the GPS antenna. This laboratory calibration was fine-tuned using the measured data, e.g., by utilizing the data acquired by driving the same location in two directions and using some control targets. These fine-tuned calibration values were applied in recomputing the data, and in producing the second set of point clouds for analysis.

**Figure 11.**
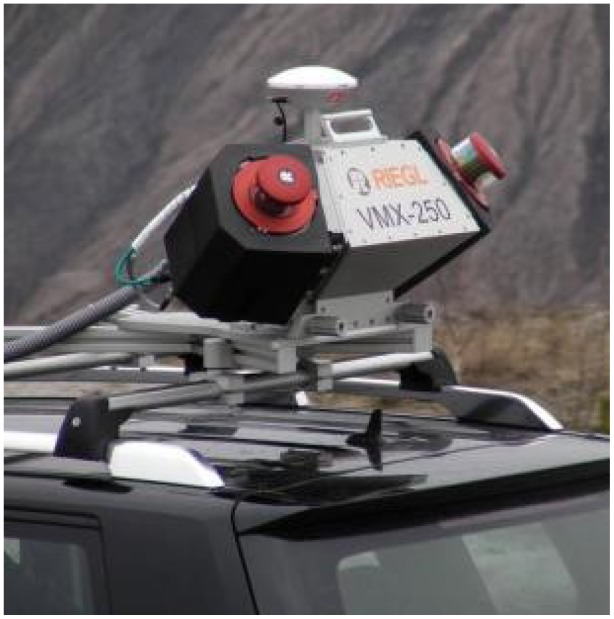
The RIEGL VMX-250 [38].

The RIEGL VMX-250 (Figure 11) was introduced at the beginning of 2010 and the test field data were acquired in March 2010. The system consists of two RIEGL VQ-250 scanners and a navigation unit with IMU, GNSS and odometer instruments. Each of the scanners measures up to 300,000 points and 100 profiles per second. The maximum measurement range is 500 m. In September 2011, RIEGL announced another mobile mapping system, the VMX-450, with VQ-450 scanners, and the capability to measure up to 550,000 points and 200 profiles per second.

RIEGL delivered two point clouds for analysis. The first batch of data was received in June 2010. Later on, RIEGL announced that they have developed their system calibration further and wish to implement their latest expertise also in the test field data. A small set of control points from the test field was delivered to RIEGL to assist in the calibration procedure. The second batch of data was received in May 2011.

The FGI Sensei is a low-cost modular measurement system consisting of a number of measurement instruments. These include a GPS/IMU positioning system, two laser scanners, a CCD camera, a spectrometer and a thermal camera.

The above GPS/IMU system is a NovAtel SPAN-CPT integrated GPS/INS receiver system, embodying NovAtel's OEMV GNSS precision receiver technology with three fiber optic gyros and three MEMS accelerometers in a single unit. The SPAN-CPT delivers 3D position, velocity and attitude solutions. The measurements of the different INS subsystems are combined using Waypoint Inertial Explorer and GPS virtual reference station (VRS) data.

**Figure 12.**
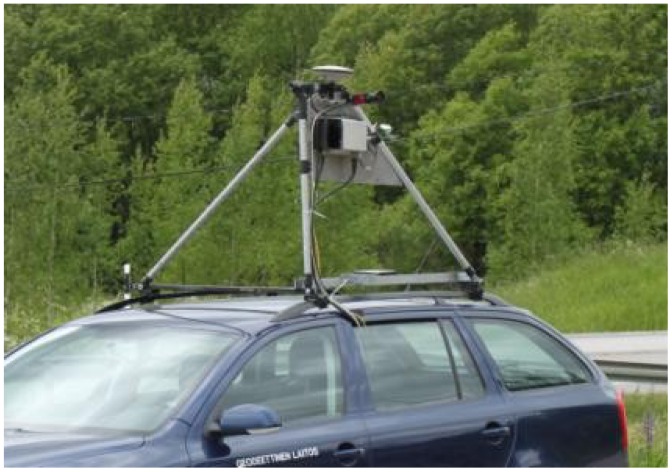
The Sensei mounted on the roof of a car.

An Ibeo Lux laser scanner was used on the Espoonlahti test field. The Ibeo Lux simultaneously measures points from four different layers and it is theoretically capable of measuring up to 38,000 points/second if only one return per pulse per layer is assumed. The scanner is able to record up to three returns per pulse per layer, thus enabling it to get hits from building walls or the ground even when these are covered by nearby trees or other vegetation. Its distance measurement range is 0.3 m to 200 m (50 m for targets with 10% remission), its ranging accuracy is 10 cm, its angular resolution is 0.25° and the divergence of its laser beam is 1.4 mrad horizontally and 14 mrad vertically with respect to the scanner body, meaning that objects may appear elongated in the vertical direction. When mounted on the roof of a car (Figure 12), this vertical elongation turns into a horizontal error as the scanner is mounted vertically. For this reason, most of the reference targets for analysis of planimetric accuracy could not be reliably measured, and the performance analysis was completed only for elevation accuracy. When the Sensei is used on the roof of a car, the instruments point towards the side of the car, and the laser scanner scans vertically, and thus covers only one side of the trajectory at a time [39].

**Figure 13.**
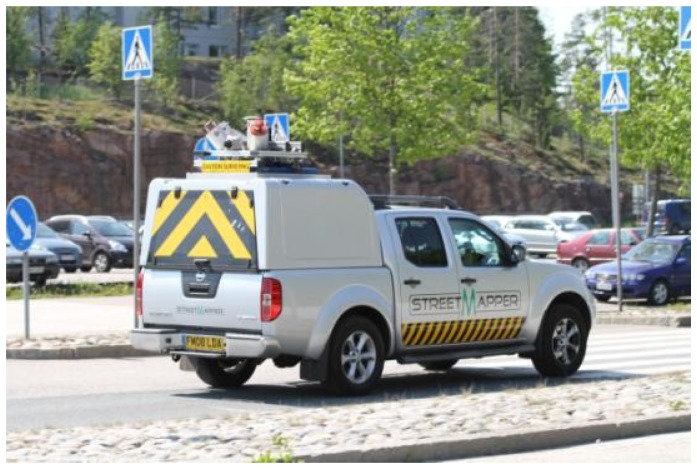
The Streetmapper 360 mapping the roads on the Espoonlahti test field.

The Streetmapper 360 (Figure 13) was launched in October 2008 and test site data were acquired in June 2011. The system consists of two RIEGL VQ-250 scanners and a navigation unit with a fiber-optic gyro-based IMU, GPS and Direct Inertial Aiding (DIA) to assist in areas of poor GPS reception. Each scanner measures up to 300,000 points and 100 profiles per second. The maximum measurement range is 500 m.

**Figure 14.**
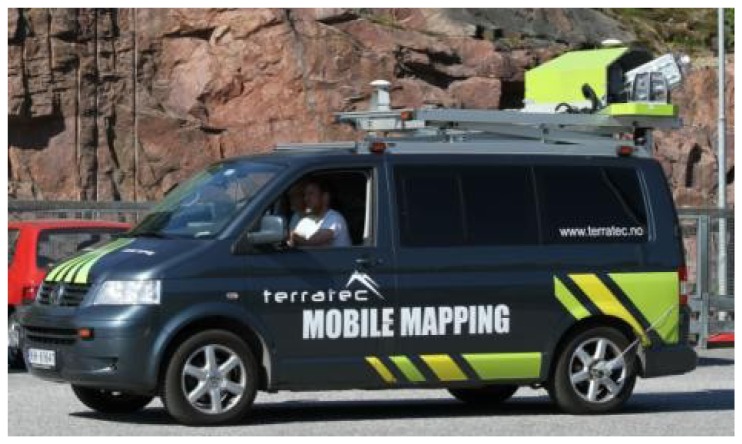
TerraTec AS mapping the Espoonlahti test field with an Optech Lynx Mobile Mapper.

The Optech Lynx Mobile Mapper was introduced in September 2007. The test field data were collected in June 2011 by TerraTec AS (Figure 14) using a system with two Optech scanners, which produce 200,000 points per second each with ranging up to 200 m. The navigation system is an Applanix POS LV 420 system with IMU, GPS and odometer instruments. The newest version Lynx Mobile Mapper M1 can produce up to 500,000 points per second per scanner.

### 2.2. Methods for Accuracy Evaluation

Firstly, the received point clouds were checked by comparing them with the reference data (Section 2.1.2) to detect any gross errors either in elevation or plane. If there was a larger systematic shift than one of a few centimeters, this was compensated to ensure validity in the comparison; especially a large systematic shift in plane can lead to distorted elevation accuracy results and it is a common practice to use some ground control points in laser scanning surveys to eliminate the bias.

Comparison between the elevation reference points and the received MLS point clouds was carried out using the *Output control report* tool in TerraScan-software [40]. It reads in the reference points and loads every laser point within a given search radius from the individual reference points. Then a small triangulated surface model is created from the laser points and laser elevation is computed for each of the reference points' easting-northing locations from the triangulated model surface. This effectively interpolates the laser elevation from the three laser points closest to the reference point to be compared. The search radius used for the densest point clouds (RIEGL, Streetmapper and Optech Lynx) was 20 cm, for the others it was 50 cm. The maximum permitted slope in the triangulated model was set to 20°.

The planimetric accuracy was evaluated by measuring the reference targets in the received MLS point clouds and then computing the differences in easting and northing.

The most deviating values were checked against the ground truth and removed from the analysis if there was any doubt that the error was due to the target, not due to the system. These errors were mainly detected in the analysis of elevation accuracy and they were due to parked cars or changes in vegetation. Following this ‘gross error filtering’, the systematic errors were removed, in plane separately for easting and northing, and the accuracy values were computed. The minimum, maximum and standard deviation values were computed for both elevation and planimetric accuracy. The mean and root mean squared errors (abbreviated to RMSE, Equation (1)) were determined for the description of the planimetric accuracy:

(1) $$\text{RMSE}=\sqrt{\frac{\overset{n}{\underset{i=1}{\text{∑}}}{\left(d_{i}\right)}^{2}}{n}}$$

where *d_i_* is the distance between the reference target and the point cloud target, and *n* is the number of samples.

## 3. Results

### 3.1. Elevation Accuracy

**Figure 15.**
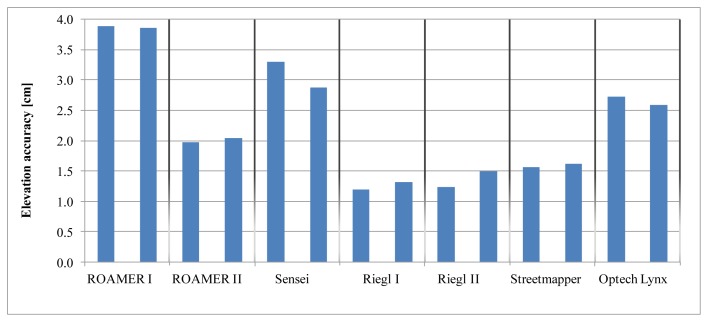
The elevation accuracy values (std), in cm, for the tested MLS systems for the two driving directions. The left column stands for counter-clockwise, CCW, and the column on the right for clockwise, CW, direction.

The results of the analysis of elevation accuracy are shown in Table 3 and Figure 15. Two results are given for the ROAMER and the RIEGL, Column I before fine-tuning and Column II after fine-tuning (see Section 2.1.3 for details).

**Figure 16.**
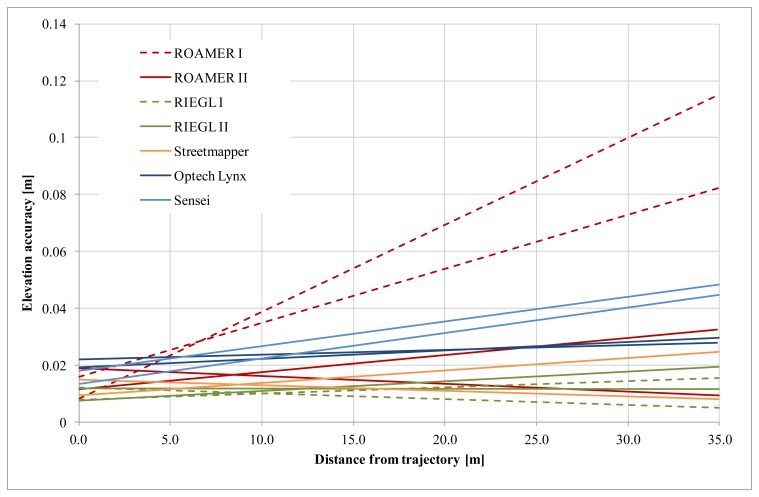
Elevation accuracy as a function of distance from the trajectory; with linear trend lines fitted to the observed errors in the two driving directions.

The RIEGL data were acquired while there still was a lot of snow on the ground, and so the number of reference points that could be used in the RIEGL analysis was lower than when using the other high-density systems. The Sensei covered only one side of the trajectory, which explains the lower number of used reference points. Elevation accuracy as a function of distance from the trajectory is shown in Figure 16. Fine-tuning has a significant effect on the ROAMER system performance.

Even though Figure 16 suggests that the elevation accuracy improves in some cases when the distance from the trajectory increases, this is unlikely to be so. This phenomenon is most propably caused by the accuracy of the reference data having reached its limits and not being available for analysis of sub-centimeter accuracy. Nonetheless, this proves that the elevation accuracy of the best MLS systems can reach values of 1–2 cm up to a range of 35 m.

### 3.2. Planimetric Accuracy

**Figure 17.**
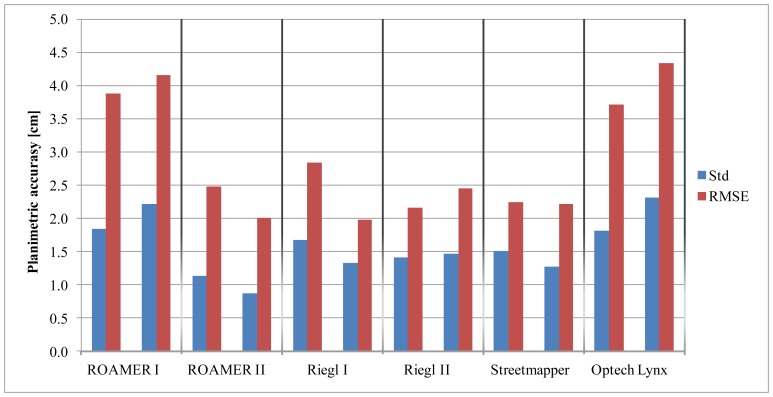
The planimetric accuracy of the tested MLS systems in two driving directions. The left column stands for counter-clockwise, CCW, and the one on the right for clockwise, CW, direction.

The results of analysis of planimetric accuracy are shown in Table 4 and Figure 17. Two results are given for the ROAMER and the RIEGL, Column I before fine-tuning and Column II after fine-tuning (see Section 2.1.3 for details).

**Figure 18.**
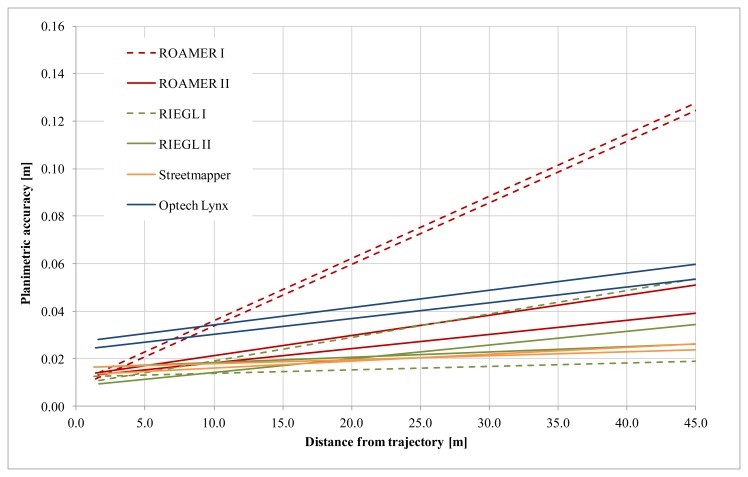
Planimetric accuracy as a function of distance from the trajectory; with the linear trend lines fitted to the observed errors in the two driving directions.

The planimetric accuracy was not analyzed for the Sensei as the reference targets could not be reliably measured from the Sensei point clouds (details in Section 2.1.3). Planimetric accuracy as a function of distance from the trajectory is shown in Figure 18. Fine-tuning has a significant effect on the ROAMER system's performance.

With properly calibrated high-end MLS systems, the planimetric accuracy in good GNSS conditions is high, and in the present study it was within the limits of the used reference data accuracy, *i.e.*, about 2 cm. Accuracy deteriorates as the distance to the trajectory increases, but very moderately, when the system calibration is in order.

The results for the ROAMER in Figures 16 and 18 show very clearly how accuracy is affected when there are problems with calibration (ROAMER I) and how re-calibration using collected point cloud data can improve the performance (ROAMER II). Similar performance improvement can be expected when errors in trajectory, caused by satellite signal outtakes or IMU disturbances, for example, are compensated for using strip adjustments or control targets, for instance.

## 4. Discussion

As the results show, the tested MLS systems are capable of acquiring accurate point cloud data under good GNSS coverage conditions. Buildings, trees and other structures often cause disturbances in satellite visibility. Moreover, the performance of other navigation instruments, such as IMUs and odometers as well as post-processing algorithms, defines the achievable accuracy. Tools for trajectory accuracy improvement are being developed and new satellites are being launched, which should improve accuracy in areas where the current systems have problems.

Even though the computation of the sensor's driving path and orientation results in observation (GNSS, IMU) errors being minimized, there are still errors in laser distance measurement, in scanning mirrors, in position (GNSS) and in orientation (IMU). Consequently, there are systematic offsets and random variations both in plane and height [41]. These errors can be minimized by means of strip adjustment, which is earlier known from ALS (e.g., TerraMatch), and which requires repeated measurements of the same surfaces and objects. With MLS, the possible objects and surfaces, which can be used in the correction process, include elevation model, paintings in the pavement, vertical poles and building corners.

**Figure 19.**
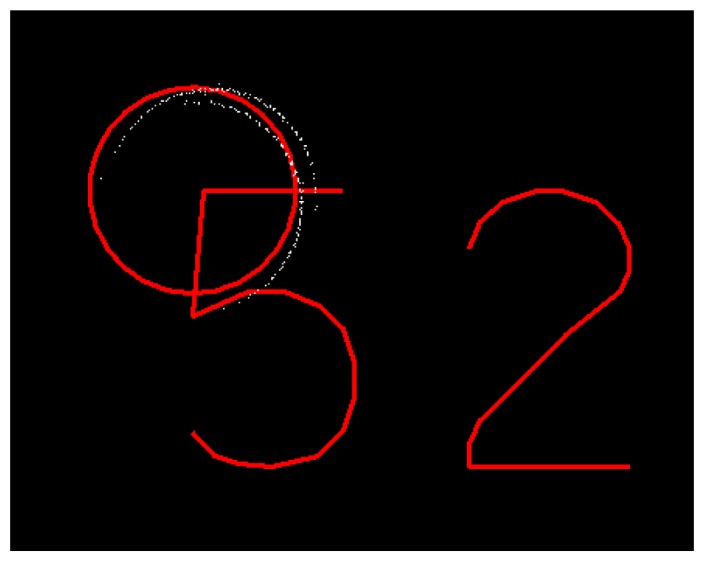
A large pole (reference target no. 52) seen double by a dual-scanner system (white points).

Imperfect boresight calibration, in addition to the navigation errors, between the scanners on a multi-scanner systems leads to multiple reproductions of objects, as in Figure 19. These kinds of errors in the relative orientation of the instruments lead to errors in the measured point clouds, which can cause problems in the continued processing of the data, such as extraction and modeling of objects. Systematic offset errors between the sensors (ΔX, ΔY, ΔZ) can be detected using observations of common objects close to them. For example, paintings on the road surface are feasible for such analysis. The Δroll error can be detected using the elevation model acquired with multiple surveys; Δpitch and Δheading errors can be detected with vertical objects such as poles and building corners. These systematic errors can be corrected appropriately by even manual processing of the data. The time-dependent variation of these data (random part) needs larger numbers of observations for corrections, which calls for the development and use of more automated techniques.

Since the results produced by all of the systems were adequate for high level of roadside mapping, the main focus in MLS quality research should be on the improvement of the trajectory solution under non-ideal (and also with ideal) conditions using both improved hardware (additional sensors) and software solutions (post-processing). As regards the hardware, the commercially available systems already include odometers. Additional sensors improving the georeferencing/localization solution during GNSS signal loss should be included. Examples of these additional sensors are cameras, whose data are automatically processed to solve the position changes together with IMU and odometer when GNSS signal is not appropriate. Alternative solutions include the use of signals, e.g., painted signals on the road surfaces, as has been demonstrated by Soininen [42] in connection with the Helsinki tram MLS survey.

The MLS data for comparison was obtained by applying the standard process of the data providers. Laboratory calibration of offsets and orientations of system components can be fine-tuned by the real-live data. In Optech and Streetmapper both of these calibrations are assumed to have been done already by the data provider. With RIEGL and ROAMER, the calibration was done first with by using only the laboratory-type calibration, which showed that there was a need for further calibration in the field. The second point cloud was provided taking the aspects mentioned in Section 2.1.3 into account, and (for example) in ROAMER a significant roll error was found with overlapping point cloud data. For future reference, the improvement of georeferencing solutions via MLS strip adjustment process needs more automation. The corrections to the offsets and orientations need to be made to the trajectory data.

The improvements in georeferencing could also include improvements due to abrupt jumps and other errors in the data. Schwarz and El-Sheimy [43] have discussed the use of three post-processing techniques, such as denoising, auto regression modeling and smoothing. Denoising is noise reduction done directly to the sensor measurements. Auto regression modeling reduces noise in the data fusion step, and forward and backward Kalman filters are example of this method type and they have already been implemented in the typical GNSS/IMU integration. Also, numerical smoothing on the post-processed trajectories can be performed.

## 5. Conclusions

It was within the context of the European Spatial Data Research project “Mobile Mapping—Road Environment Mapping Using Mobile Laser Scanning” that most of the leading mobile mapping manufactures, among them RIEGL, Optech and Streetmapper, together with some research systems participated in the benchmarking of the performance of mobile laser scanning systems. The verification was done using a permanent test field established in Espoonlahti (Finland). The comparison revealed that high-quality point clouds can be generated by all systems under good GNSS conditions. With all professional systems properly calibrated, the elevation accuracy was better than 3.5 cm up to a range of 35 m. The best system had a planimetric accuracy of 2.5 cm even with range of 45 m. The planimetric error increases as a function of range, but moderately so if the system is properly calibrated. Proper calibration can be achieved, for example, by using the test field concept also for mobile laser scanning. The main focus in mobile laser scanning development in the near future should be on the improvement of the trajectory solution, especially under non-ideal conditions, using both improvements in hardware and computational solutions.

## Figures and Tables

**Table 1.** The tested MLS-systems.

**MLS system**	**Operated by**	**Data acquisition date**
ROAMER	Finnish Geodetic Institute	June 2009
RIEGL VMX-250	RIEGL Laser Measurement Systems GmbH	March 2010
Sensei	Finnish Geodetic Institute	May 2011
Streetmapper 360	3D Laser Mapping	June 2011
Optech Lynx	TerraTec AS	June 2011

**Table 2.** The MLS scanner specifications [32–37].

	**Optech Lynx Mobile Mapper/TerraTec AS**	**Sensei**	**ROAMER**	**RIEGL VMX-250 and Streetmapper 360**
Laser scanner	Optech	Ibeo Lux	FARO Photon 80	RIEGL VQ-250
Laser wavelength	N/A	905 nm	785 nm	Near infrared
Distance measurement principle	Time-of-flight, max four returns	Time-of-flight, max three returns	Phase-shift	Time-of-flight, no. of returns selectable
Points/s (×1000) max	2 × 200	38	120	2 × 300
Range	200 m	200 m	76 m	500 m
Profiles/c max	2 × 200	50	61	2 × 100
Beam divergence	N/A	1.4 × 14 mrad	0.16 mrad	0.3 mrad
Beam size at exit	N/A	N/A	3.3 mm	7 mm
Distance measurement accuracy	8 mm	100 mm	2 mm@25 m	10 mm@150 m
Angular resolution	N/A	0.25°	0.009°	0.018°

**Table 3.** The elevation accuracy values, in cm, for the tested MLS systems. Driving directions: counter-clockwise CCW and clockwise CW. N is the number of used reference points.

	**ROAMER I**		**ROAMER II**		**Sensei**		**RIEGL I**		**RIEGL II**		**Streetmapper**		**Optech Lynx**	
	**CCW**	**CW**	**CCW**	**CW**	**CCW**	**CW**	**CCW**	**CW**	**CCW**	**CW**	**CCW**	**CW**	**CCW**	**CW**
N	2,936	2,946	2,819	2,816	1,418	1,450	1,566	1,600	1,585	1,585	2,790	2,549	2,693	2,527
Min	−12.9	−17.1	−6.8	−7.5	−11.1	−10.1	−5.7	−5.3	−4.6	−5.7	−5.0	−5.0	−7.1	−6.4
Max	10.4	12.6	6.0	5.0	14.3	15.0	6.1	4.1	6.5	5.2	5.4	5.1	8.1	9.1
Std	3.9	3.9	2.0	2.0	3.3	2.9	1.2	1.3	1.2	1.5	1.6	1.6	2.7	2.6

**Table 4.** The planimetric accuracy values, in cm, for the tested MLS systems. Driving directions: counter-clockwise CCW and clockwise CW. N is the number of used reference targets.

	**ROAMER I**		**ROAMER II**		**RIEGL I**		**RIEGL II**		**Streetmapper**		**Optech Lynx**	
	**CCW**	**CW**	**CCW**	**CW**	**CCW**	**CW**	**CCW**	**CW**	**CCW**	**CW**	**CCW**	**CW**
N	124	120	136	120	169	178	193	192	190	177	160	160
Mean	3.4	3.5	2.2	1.8	2.3	1.5	1.6	2.0	1.7	1.8	3.2	3.7
Min	0.4	0.5	0.2	0.3	0.1	0.0	0.1	0.0	0.0	0.2	0.1	0.3
Max	8.7	11.8	6.7	5.1	11.7	11.7	11.5	10.6	9.4	9.7	12.3	13.2
Std	1.8	2.2	1.1	0.9	1.7	1.3	1.4	1.5	1.5	1.3	1.8	2.3
RMSE	3.9	4.2	2.5	2.0	2.8	2.0	2.2	2.5	2.2	2.2	3.7	4.3

## References

[1] Barber D., Mills J., Smith-Voysey S. (2008). Geometric validation of a ground-based mobile laser scanning system. ISPRS J. Photogramm. Remote Sens..

[2] Brenner C. (2009). Extraction of features from mobile laser scanning data for future driver assistance systems. Adv. GIScience.

[3] Clarke K.C. (2004). Mobile mapping and geographic information systems. Cartogr. Geogr. Inform..

[4] El-Sheimy N. An Overview of Mobile Mapping Systems.

[5] Früh C., Zakhor A. (2004). An automated method for large-scale, ground-based city model acquisition. Int. J. Comp. Vis..

[6] Graham L. (2010). Mobile mapping systems overview. Photogramm. Eng. Remote Sens..

[7] Haala N., Peter M., Kremer J., Hunter G. (2008). Mobile LiDAR mapping for 3D point cloud collection in urban areas—A performance test. Int. Arch. Photogramm. Remote Sens. Spatial Inf. Sci..

[8] Hassan T., El-Sheimy N. (2008). Common adjustment of land-based and airborne mobile mapping system data. Int. Arch. Photogramm. Remote Sens. Spatial Inf. Sci..

[9] Jaakkola A., Hyyppä J., Hyyppä H., Kukko A. (2008). Retrieval algorithms for road surface modelling using laser-based mobile mapping. Sensors.

[10] Kukko A. (2009). Road Environment Mapper—3D Data Capturing with Mobile Mapping. Licentiate's Thesis.

[11] Kukko A., Andrei C.-O., Salminen V.-M., Kaartinen H., Chen Y., Rönnholm P., Hyyppä H., Hyyppä J., Chen R., Haggrén H., Kosonen I., Čapek K. Road Environment Mapping System of the Finnish Geodetic Institute—FGI Roamer.

[12] Kukko A., Jaakkola A., Lehtomäki M., Kaartinen H., Chen Y. Mobile Mapping System and Computing Methods for Modelling of Road Environment.

[13] Kukko A., Kaartinen H., Hyyppä J., Chen Y. (2012). Multiplatform Mobile laser scanning: Usability and performance. Sensors.

[14] Kukko A., Hyyppä J. (2009). Small-footprint laser scanning simulator for system validation, error assessment and algorithm development. Photogramm. Eng. Remote Sens..

[15] Lehtomäki M., Jaakkola A., Hyyppä J., Kukko A., Kaartinen H. (2010). Detection of vertical pole-like objects in a road environment using vehicle-based laser scanning data. Remote Sens..

[16] Manandhar D., Shibasaki R. (2002). Auto-extraction of urban features from vehicle-borne laser data. ISPRS J. Photogramm. Remote Sens..

[17] Petrie G. (2010). Mobile mapping systems: An introduction to the technology. GeoInformatics.

[18] Shen Y., Sheng Y., Zhang K., Tang Z., Yan S. Feature Extraction from Vehicle-Borne Laser Scanning Data.

[19] Steinhauser D., Ruepp O., Burschka D. Motion Segmentation and Scene Classification from 3D LIDAR Data.

[20] Tao C., Li J. (2007). Advances in Mobile Mapping Technology.

[21] Weiss T., Dietmayer K. Automatic Detection of Traffic Infrastructure Objects for the Rapid Generation of Detailed Digital Maps Using Laser Scanners.

[22] Yu S.J., Sukumar S.R., Koschan A.F., Page D.L., Abidi M.A. (2007). 3D reconstruction of road surfaces using an integrated multi-sensory approach. Opt. Lasers Eng..

[23] Zhao H., Shibasaki R. (2003). Reconstructing a textured CAD model of an urban environment using vehicle-borne laser range scanners and line cameras. Mach. Vis. Appl..

[24] Zhao H., Shibasaki R. (2003). A vehicle-borne urban 3-D acquisition system using single-row laser range scanners. IEEE T. Syst. Man Cybern. B..

[25] Zhao H., Shibasaki R. (2005). Updating a digital geographic database using vehicle-borne laser scanners and line cameras. Photogramm. Eng. Remote Sens..

[26] Narayana K. (2011). Solutions for the localization of Mobile Mapping Systems in Structured Environments. Ph.D. Thesis.

[27] Kaartinen H., Hyyppä J. (2006). EuroSDR Project, Commission III “Evaluation of Building Extraction”; Final Report.

[28] Kaartinen H., Hyyppä J. (2008). EuroSDR/ISPRS Project, Commission II “Tree Extraction”; Final Report.

[29] Yen K.S., Akin K., Lofton A., Ravani B., Lasky T.A. (2010). Using Mobile Laser Scanning to Produce Digital Terrain Models of Pavement Surfaces.

[30] Bilker M., Kaartinen H. (2001). The Quality of Real-Time Kinematic (RTK) GPS Positioning.

[31] Häkli P. Practical Test on Accuracy and Usability of Virtual Reference Station Method in Finland.

[32] (2008). FARO Photon 80 specifications.

[33] (2009). FARO Photon 120 specifications.

[34] Ibeo Lux specifications. http://www.ibeo-as.com/.

[35] Lynx Mobile Mapper Spec Sheet. http://www.optech.ca/pdf/Lynx_SpecSheet_110909_web.pdf.

[36] Datasheet RIEGL VQ-250. http://riegl.com/uploads/tx_pxpriegldownloads/10_DataSheet_VQ250_13-04-2010.pdf.

[37] TerraTec. (2007). Mobile Mapping.

[38] RIEGL image gallery. http://www.riegl.com/nc/products/mobile-scanning/gallery/.

[39] Jaakkola A., Hyyppä J., Kukko A., Yu X., Kaartinen H., Lehtomäki M., Lin Y. (2010). A low-cost multi-sensoral mobile mapping system and its feasibility for tree measurements. ISPRS-J. Photogramm. Rem. Sens..

[40] TerraScan User's Guide 03.10.2005. http://www.terrasolid.fi.

[41] Pfeifer N., Oude Elberink S., Filin S. (2005). Automatic Tie Elements Detection for Laser Scanner Strip Adjustment. International Archives of Photogrammetry, Remote Sensing and Spatial Information Sciences.

[42] Soininen A. Presentation in Terrasolid European User's Event 2012 in Levi, Finland. http://www.terrasolid.fi/system/files/helsinki_tram_survey.ppt.

[43] Schwarz K.P., El-Sheimy N. (2004). Mobile Mapping Systems – State of The Art and Future Trends. International Archives of Photogrammetry, Remote Sensing and Spatial Information Sciences.

